# Novel *Bacillus ginsengihumi* CMRO6 Inhibits Adipogenesis via p38MAPK/Erk44/42 and Stimulates Glucose Uptake in 3T3-L1 Pre-Adipocytes through Akt/AS160 Signaling

**DOI:** 10.3390/ijms23094727

**Published:** 2022-04-25

**Authors:** Kyung Dong Lee, Soundharrajan Ilavenil, Muthusamy Karnan, Chul-Ju Yang, Dahye Kim, Ki Choon Choi

**Affiliations:** 1 Department of Companion Animals, Dongsin University, Naju 58245, Korea; leekd@dsu.ac.kr; 2 Grassland and Forages Division, National Institute of Animal Science, Rural Development Administration, Cheonan 31000, Korea; ilavenil@korea.kr (S.I.); karnantm111@gmail.com (M.K.); 3 Department of Animal Science and Technology, Sunchon National University, Suncheon 57922, Korea; yangcj@scnu.kr; 4 Animal Genomics and Bioinformatics Division, National Institute of Animal Science, Wanju 55365, Korea; dhkim0724@korea.kr

**Keywords:** *Bacillus ginsengihumi*, probiotic, cell-free metabolites, 3T3-L1, lipid, glucose uptake

## Abstract

The health benefits of probiotics have been known for decades, but there has only been limited use of probiotics in the treatment of obesity. In this study, we describe, for the first time, the role of cell-free metabolites (CM) from *Bacillus ginsengihumi*-RO6 (CMRO6) in adipogenesis and lipogenesis in 3T3-L1 pre-adipocytes. The experimental results show that CMRO6 treatment effectively reduced lipid droplet accumulation and the expression of CCAAT/enhancer-binding protein α and β (C/EBPα and C/EBPβ), peroxisome proliferator-activated receptor γ (PPAR-γ), serum regulatory binding protein 1c (SREBP-1c), fatty acid-binding protein 4 (FABP4), fatty acid synthase (FAS), acetyl CoA carboxylase (ACC), phosphorylated p38MAPK, and Erk44/42. Additionally, CMRO6 treatment significantly increased glucose uptake and phosphorylated Akt (S473), AS160, and TBC1D1 protein expressions. Considering the results of this study, *B. ginsengihumi* may be a novel probiotic used for the treatment of obesity and its associated metabolic disorders.

## 1. Introduction

Globally, obesity is considered a global health threat. Its incidence rates have increased worldwide in recent years. It is estimated that the population obesity percentage in China is 10.7%, 12.8% in the European countries, and 30.4% in the United States [1,2,3]. Moreover, a world health organization (WHO) data prediction indicates that 39% of people in the world today will likely develop obesity by 2035 [4]. In Korea, more than 4% of the adult population are obese, and approximately 30% of adults are overweight, according to the organization for Economic Co-operation and Development (OECD). A study published by Yoon-Sun Jung et al., 2020 reported that the median body mass index (BMI) for Korean adults in 2040 is expected to be around 23.55 kg/m^2^. It is estimated that 70.05% of adults will be obese by 2040, according to BMI classification [5]. Increasing obesity burdens health care and economic systems due to its close association with several chronic diseases, such as cardiovascular diseases, aging, diabetes, cancer, musculoskeletal illnesses, inflammatory issues, and fatty liver, etc. [6,7]. The number of deaths among adults across the globe has been increasing every year [8]. A number of factors contribute to the development of obesity, such as a sedentary lifestyle, high caloric intake, depression, as well as various social and monetary issues. One common factor is fat deposition within adipocytes during adipogenic and lipogenic processes [9]. The accumulation of lipids in adipocytes occurs through multiple complex process and involves several genes and transcriptional factors, such as the CCAAT/enhancer-binding protein family (C/EBPs) and peroxisome proliferator-activated receptor γ (PPAR-γ), which induce the expression of lipogenic genes, such as fatty acid synthase, acetyl CoA carboxylase (ACC), fatty acid-binding protein 4 (FABP4) [10,11,12]. Hence, several researchers have focused on the development of dietary supplements that can effectively quell excess fat deposition in adipocytes with minimal effects. In fact, probiotics play a major role in preventing obesity, which has been proven in several recent studies [13,14,15]. To date, several molecular mechanisms have been identified as underlying anti-obesity effects of probiotics. These mechanisms include metabolic energy changes, improvements in intestinal barrier metabolism, immune response, and modulation of nerve activity and appetite [13,15,16,17]. Bacillus species have been shown to exert a significant effect on metabolic disorders [18]. For example, *B. licheniformis* reduced body weight gain, fat accumulation, and improved glucose tolerance in animals that were induced to gain weight via obesity [19,20,21,22], and *B. coagulans* significantly improved bile acid metabolic dysfunction and NAFLD in rats fed a diet that contained a high concentration of cholic acid supplement [23]. The exopolysaccharide produced by *Bacillus subtilis* reduced the level of serum glucose and cholesterol in diabetic animals [24]. Furthermore, *B. subtilis* inhibits the differentiation of adipocytes and the accumulation of lipids in 3T3-L1 cells by downregulating key transcriptional factors and signaling pathways [25]. *Bacillus ginsengihumi* is a Gram-positive, facultatively aerobic, non-motile bacterial strain that forms endospores during growth; it also acts as a good biocontrol agent [26,27]. However, *Bacillus ginsengihumi* effects on adipocyte differentiation and lipid accumulation in 3T3-L1 adipocytes have not yet been explored. In this respect, the novel *B. ginsengihumi* was isolated and characterized. Additionally, the impact of extracellular metabolites produced by *B. ginsengihumi* on the differentiation of adipocytes, lipid accumulation, and molecular pathways were also investigated in 3T3-L1 cells.

## 2. Results

### 2.1. Cytotoxic Effects of Cell-Free Metabolites from *B. ginsengihumi* (CMRO6) on 3T3-L1 Pre-Adipocytes

**Figure 1:**
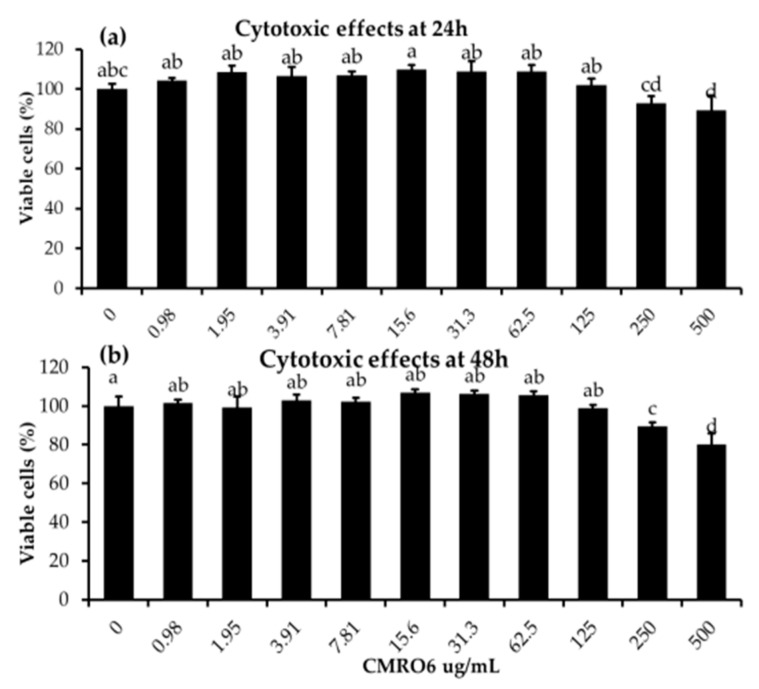
Cytotoxic effects of cell-free metabolites of *B. ginsengihumi*-RO6 (CMRO6) on 3T3-L1 pre-adipocytes. The cells were treated with different concentrations of CMRO6 (0.98–500 μg/mL) and incubated under normal cell culture conditions. After that, cell viabilities were determined after 24 h and 48 h using EZ-cytox reagent. (**a**) The percentage of viable cells in the experimental groups at 24 h; (**b**) the percentage of viable cells in the experimental groups at 48 h. The data are presented as the mean ± STD for six replicates (n = 6). Different letters within the figure indicate significant differences between groups (*p* < 0.05).

Pre-adipocytes were seeded in 96-well cell culture plates and treated with different concentrations of CMRO6 (0.98 µg/mL–500 µg/mL) for 24 and 48 h. It was found that CMRO6 concentrations between 0.98 and 125 µg/mL did not affect cellular morphology or viability, while doses above 125 µg/mL had slight toxic effects on pre-adipocytes at both 24 and 48 h (Figure 1).

### 2.2. Differentiation and Lipid Accumulation

**Figure 2:**
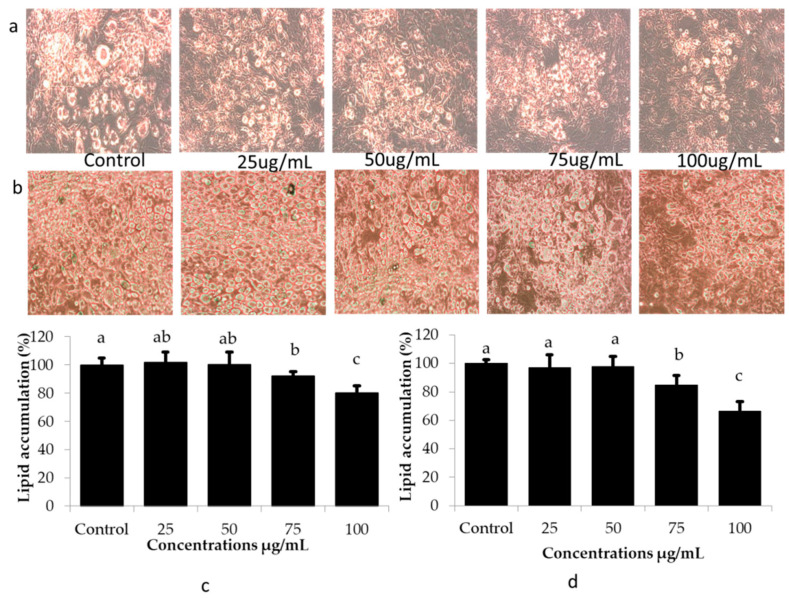
Impact of CMRO6 at different concentrations on lipid accumulation and differentiation. Cells were seeded in cell culture plates and incubated at 37 °C with 5% CO_2_. The differentiation was induced by dexamethasone, IBMX, and insulin for 48 h and then media was replaced by insulin medium for another 48 h. CMRO6 at different concentrations was added to the cell when differentiation began. Differentiated cells were then monitored under an Evos microscope and lipid deposits were stained with Oil Red O stain. The differentiated cells were photographed on the 5th and 10th day of differentiation. The stained lipids were extracted using isopropyl alcohol and lipid levels were measured. (**a**,**b**) Microscopic views of lipid accumulation in differentiated cells at 5th and 10th day of differentiation; (**c**,**d**) the percentage of lipids in experimental adipocytes at 5th and 10th day of differentiation. The data are represented as the mean ± STD of six replicates (n = 6). Different letters within the figure indicate significant differences between groups (*p* < 0.05).

The effects of CMRO6 at various concentrations (25–100 g/mL) on the differentiation and lipid accumulation in adipocytes on days 5 and 10 were monitored and determined using light microscopy and Oil Red O staining, respectively. Microscopy revealed that differentiation cocktails (IBMX, insulin, and dexamethasone) induced rapid differentiation and lipid accumulation in 3T3-L1 adipocytes. Control cells had several large lipid droplets in a number of regions of the differentiated adipocytes, while treatment with CMRO6 reduced the size and number of lipid-accumulated regions dose-dependently (Figure 2a,b). Furthermore, it was confirmed by the Oil Red O staining (ORO) method, that the low concentrations (25 to 50 µg/mL) of CMRO6 failed to inhibit differentiation cocktail-induced lipid accumulation significantly. CMRO6 at 75 µg/mL concentration showed moderate inhibitory activities at a significant level (*p* < 0.05), but at 100 µg/mL, CMRO6 strongly reduced the number and size of lipids deposition compared with the control and other concentrations. The ORO stain was extracted from experimental cells using 100% isopropyl alcohol, and the intensity was measured at 450 nm. Lower absorbance was subsequently observed in the cells treated with CMRO6 compared to the control cells. CMRO6 at a higher concentration effectively inhibited differentiation and lipid accumulation in adipocytes on day 5 and day 10 (Figure 2c,d).

### 2.3. CMRO6 Competes against Rosiglitazone (RGZ)-Induced Lipid Accumulation and PPAR-γ2 Expression

**Figure 3:**
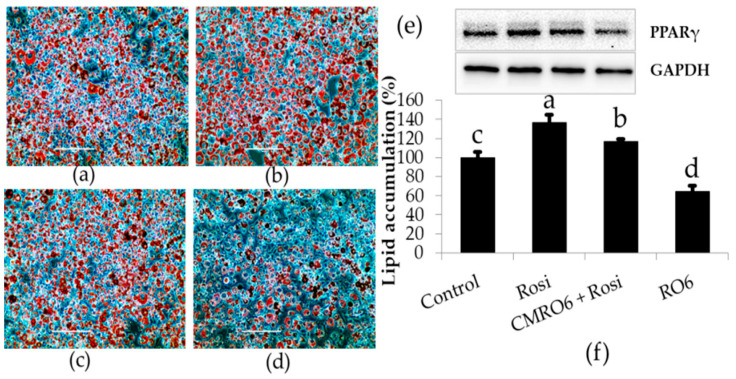
Comparative studies between rosiglitazone (RGZ) and CMRO6 on adipocyte differentiation. The cells were treated with either CMRO6 (100 ug/mL) or RGZ (1 μM), or RGZ with CMRO6, when differentiation began. Adipocytes treated with RGZ differentiated faster and accumulated more fat, while cells treated with CMRO6 showed a significant reduction in fat deposition compared to controls. CMRO6 also attenuated the RGZ-induced lipid accumulation as compared to cells treated with RGZ alone. (**a**) Control cells; (**b**) RGZ-treated cells; (**c**) RGZ + CMRO6-treated cells; (**d**) CMRO6-treated cells; (**e**) PPAR-γ protein expression in the experimental cells on day 10; (**f**) fat deposition in the experimental cells on day 10, determined by Oil Red O staining method. The data are represented as the mean ± STD of six replicates (n = 6). Different letters within the figure indicate significant differences (*p* < 0.05).

It is well known that rosiglitazone (RGZ) is a PPAR-γ agonist that induces lipid accumulation during the differentiation of adipocytes via increases in the expression of key transcriptional factor PPAR-γ. Treatment with RGZ markedly increased lipid accumulation and PPAR-γ expression compared to control cells, while treatment with CMRO6 significantly inhibited the deposition of lipid droplets and PPAR-γ expression as compared to control and RGZ-treated cells. Cells treated with CMRO6 in the presence of RGZ showed a reasonable reduction in lipid deposition level as well as PPAR-γ expression compared to those treated with RGZ alone (*p* < 0.05) (Figure 3).

### 2.4. CMRO6 Downregulates the Adipogenesis- and Lipogenesis-Related Key Markers

The microscopic analysis as well as the lipid staining study confirmed that the CMRO6 treatment effectively reduced fat deposition during the differentiation process. We further investigated the mechanism by which the CMRO6 could downregulate lipogenesis and adipogenesis.

**Figure 4:**
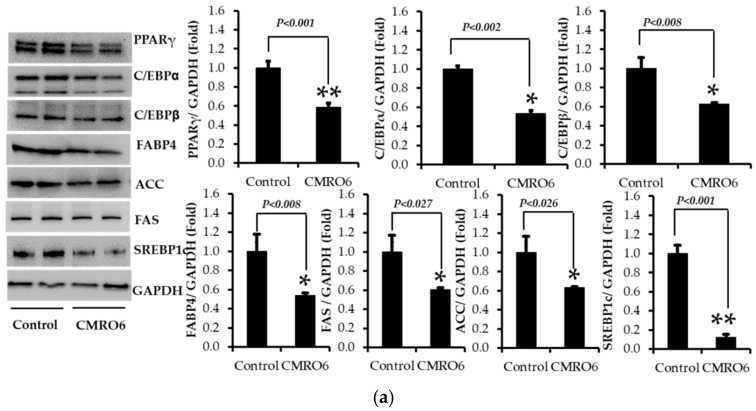

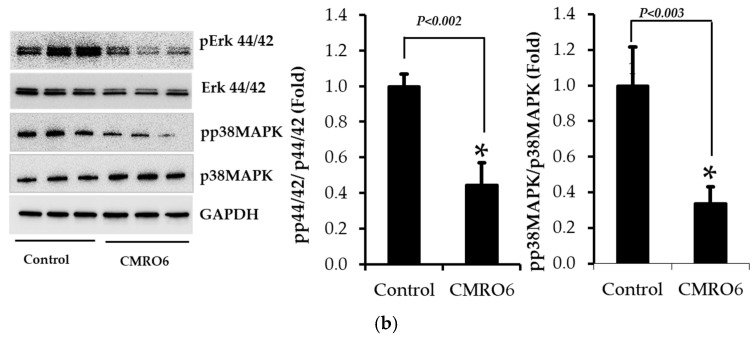
(**a**) Effect of CMRO6 on key transcriptional factors and their downstream targets. The proteins were extracted with extraction buffer on day 10 in the presence of protease and phosphatase inhibitors and quantified by the BCA method. Proteins were then separated by SDS-PAGE. The expression of C/CEBβ, C/CEBα, PPAR-γ2, SREBP-1c, FAS, ACC, and FABP4 proteins was detected with specific antibodies by the immunoblot method. The protein intensity was quantified by ImageJ software. CMRO6 treatment during differentiation reduced the translation level of C/CEBβ, C/CEBα, PPAR-γ2, SREBP-1c, FAS, ACC, and FABP4. Results are expressed as the mean ± STD, n = 3, * *p* values significant between control and treatment by an independent *t*-test. (**b**) Changes in Erk44/42 and P38MAPK signaling pathways in response to CMRO6 treatment. The proteins were extracted with extraction buffer on day 10 in the presence of protease and phosphatase inhibitors and quantified by the BCA method. Proteins were then separated by SDS-PAGE. The phosphorylating levels of Erk44/42 and p38MAPK were determined with specific antibodies by the Western blot method. The protein intensity was quantified by ImageJ software. Results are expressed as the mean ± STD of three replicates, n = 3, * *p* values (0.02-0.027), ** *p* values (0.001), control vs treatment by an independent *t*-test.

The expression of adipocyte-specific transcriptional factors, such as C/CEB-β, C/CEB-α, PPAR-γ2, and SREBP-1c, and their downstream target proteins, FAS, ACC, and FABP4, were analyzed using the Western blot method. The data from the study showed that cells treated with CMRO6 significantly downregulated the expression of C/CEBβ, C/CEBα, PPAR-γ, SREBP-1c, FAS, ACC, and FABP4 on the 10th day of differentiation compared to control cells (Figure 4a). The results confirm that the addition of CMRO6 could reduce the lipid deposition and differentiation of adipocytes through the inhibition of the expression of these key adipogenic and lipogenic proteins.

### 2.5. CMRO6 Regulates Differentiation and Lipid Accumulation via Modulating p38MAPK and Erk1/2 Signaling Pathways

A CMRO6 treatment decreased differentiation and lipid accumulation by inhibiting key proteins involved in adipogenesis and lipogenesis. Then, the signaling pathways involved in the inhibitory effect of CMRO6 on differentiation and lipid accumulation in 3T3-L1 adipocytes were determined on day 10. A significant reduction in p38MAPK phosphorylation at Thr180/Tyr182, and Erk44/42 at Thr202/Tyr204, was observed in cells treated with CMRO6 compared with control cells (Figure 4b). The data indicate that CMRO6 could modulate MAPK-dependent signaling pathways to regulate lipogenesis and adipogenesis.

### 2.6. CMRO6 Induced Glucose Uptake via Akt-Dependent Signaling Pathway

**Figure 5:**
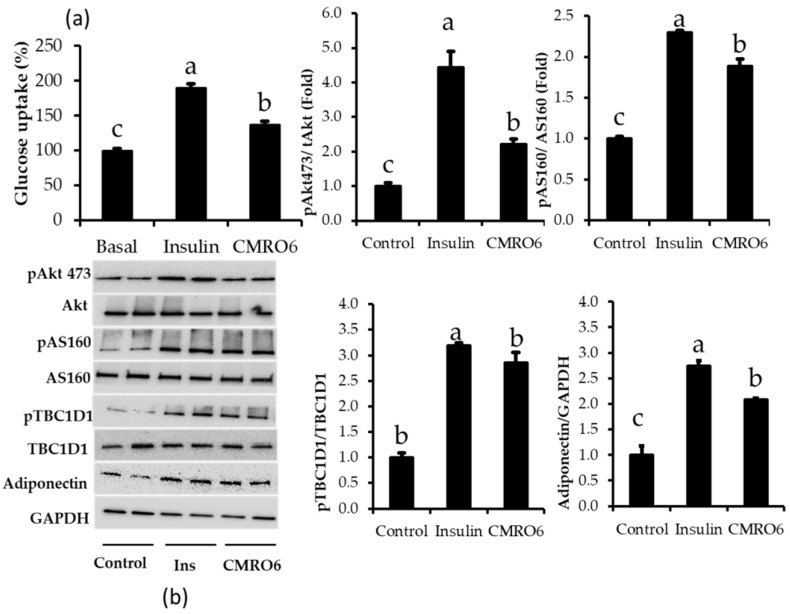
Effect of CMRO6 on glucose uptake and insulin-related signaling pathways. (**a**) Glucose uptake was measured in differentiated adipocytes with a Promega glucose uptake-Glo assay kit. (**b**) Phosphorylation levels of insulin-stimulated signaling pathways (Akt, AS160, and TBC1D1) and adiponectin expression, which is related to glucose uptake, in response to insulin and CMRO6 treatment. Results are presented as the mean ± STD of three replicates. Different letters within the figure indicate significant differences (*p* < 0.05).

CMRO6 has anti-lipidemic effects on 3T3-L1 adipocytes. Therefore, we determined whether CMRO6 has the ability to stimulate glucose uptake in differentiated adipocytes after overnight starvation; the results were compared between insulin and CMRO6 treatment (Figure 5a). 2DG/glucose uptake was rapidly accelerated by insulin treatment compared with control cells. Similarly, CMRO6 treatment increased glucose uptake by differentiated adipocytes significantly compared to control cells. However, this glucose uptake was lower than in the insulin treatment and higher than in the control cells (*p* < 0.05). Furthermore, the expression of adiponectin and the phosphorylation of Akt, at S473, and its downstream substrates (pAS160 and pTBCD-1), which are closely related to glucose uptake, were analyzed. CMRO6 and insulin treatment significantly upregulated adiponectin expression and increased phosphorylation of Akt at S473, pAS160 at Thr 642, and pTBC1D-1 at Ser 700 at a higher level than the control cells (Figure 5b).

## 3. Discussion

Scientists have become increasingly interested in exploring the role of the human microbiome (probiotics and postbiotics) in the treatment of obesity, over the last few decades [28,29]. There are several probiotics that are known to colonize epithelial cells as well as modulate key signaling molecules [30]. On the other hand, postbiotics derived from gut microbial metabolism have been broadly considered as potentially useful therapeutic agents against obesity [31]. In addition, probiotics can produce several branched-chain fatty acids that have anti-obesity properties [32]. In this study, we demonstrate, for the first time, that CMRO6 exhibits anti-obesogenic activities by inhibiting lipid deposition and differentiation in 3T3-L1 adipocytes. CMRO6 treatment led to reduced lipid droplet accumulation and decreased droplet size in adipocytes compared to control cells. Additionally, Oil Red O staining was used to determine the fat deposition level in adipocytes. As a result, we found that CMRO6 showed a significant reduction in the percent of depositions in adipocytes during differentiation in a dose-dependent manner, more so than in control cells. Our findings suggest that CMRO6 possesses anti-lipogenic and adipogenic properties in 3T3-L1 adipocytes.

As an in-vitro adipogenic model, 3T3-L1 pre-adipocytes have become an important tool for the development of new anti-obesity drugs. Several research studies have demonstrated, in response to adipogenic stimuli, increased intracellular triglyceride (TG) levels in 3T3-L1 cells, which eventually mature into white adipocytes [33,34]. Peroxisome proliferator-activated receptor γ (PPAR-γ) is a key transcriptional factor that plays an important role in the development and function of adipocytes [35]. It has been shown that activating PPAR-γ in adipocytes leads to increased fat deposition, thus reducing the amount of circulating fatty acids and increasing the synthesis of TG [36]. CCAAT/enhancer-binding protein α is another important key transcriptional factor that plays a major role in adipocyte differentiation [37]. SREBP-1c regulates both PPAR-γ and C/EBPs during the differentiation of adipocytes, and is necessary for fatty acid synthesis [38]. CMRO6 treatment significantly reduced the expression of transcription factors PPAR-γ, C/EBPα, and SREBP-1c, which indicates that CMRO6 treatment reduced adipocyte differentiation and lipid accumulation by downregulating PPAR-γ and C/EBPα expression. C/EBPβ is another important factor that is highly expressed in differentiated adipocytes. It has been shown that C/EBPβ and C/EBPδ could induce adipogenesis via the upregulation of PPAR-γ and C/EBPα [39]. The present study demonstrated that the differentiation cocktail (DC: insulin, dexamethasone, and IBMX) increased the expression of C/EBPβ protein in control adipocytes, whereas the effect of CMRO6 in the presence of DC diminished C/EBPβ protein expression. The fact that PPAR-γ and C/EBPα expressions were downregulated by CMRO6 via the inhibition of C/EBPβ provides further evidence that their expression drives lipogenesis in adipocytes.

The induction of PPAR-γ, C/EBPα, and SREBP-1c accelerates lipogenesis by the upregulation of lipogenic markers, such as FAS and ACC [40,41]. Several reports have documented that FAS, ACC, and aP2 expression increases by 10–100 fold during the terminal phase of differentiation [42,43]. There are several enzymes that are involved in fatty acid metabolism. Among these enzymes, FAS and ACC are the key enzymes responsible for fatty acid synthesis and triglyceride synthesis. A key event in lipid metabolism is the carboxylation of acetyl CoA into malonyl CoA by the acetyl CoA carboxylase (ACC). The current study found that the cells treated with CMRO6 significantly reduced the expression of lipogenic markers, such as FAS and ACC, in adipocytes, which subsequently inhibited TG synthesis, thus providing a protective effect against fat deposition in adipocytes. These results are in agreement with the Oil Red O staining of fat accumulation in control and CMRO6 treated adipocytes. SCFAs produced by probiotics exert anti-obesity effects through modulation of lipid and glucose metabolism [44,45], resulting in a decrease in adipocyte size [46,47]. In particular, SCFAs reduce fat deposition in adipose tissue by accelerating the oxidation of fatty acids [46,48] and switching the metabolic state from lipogenesis to fat oxidation through the regulation of PPAR-γ [49,50]. We found that treatment with the CMRO6 inhibited adipocyte differentiation and lipid accumulation by downregulating adipogenic and lipogenic markers. This effect may be a result of SCFA production by *Bacillus ginsengihumi*. In order to address this, we must further investigate the mechanisms by which SCFAs produced by *Bacillus ginsengihumi* modulate fat accumulation and the differentiation of adipocytes. Fatty acid-binding protein 4 (FABP4) is an adipokine that is exclusively produced in adipocytes. Elevated levels of FABP4 in the blood are associated with metabolic disorders and cardiovascular diseases. Additionally, inhibiting FABP4 secretion might also be a novel therapeutic strategy to prevent insulin resistance and type 2 diabetes [51,52]. It has been stated that adiponectin is one of the major adipokines secreted exclusively in adipocytes, and is associated with a lower incidence of diabetes [53,54]. The present study signified that the addition of CMRO6 during the adipocyte differentiation significantly reduced FABP4 expression and increased adiponectin expression compared to control cells, suggesting that CMRO6 may be able to modulate carbohydrate metabolism. It is worth noting that this statement is consistent with the glucose uptake assay results, which showed that the CMRO6 treatment enhanced glucose uptake in differentiated adipocytes. The current study confirmed that CMRO6 increased glucose uptake by inhibiting FABP4 and inhibiting adiponectin expression. Additionally, we examined the expression of the phosphorylated proteins, Akt, AS160, and TBC1D1, in experimental cells; these proteins transport glucose into the cell via the translocation of GLUT4. Akt substrate of 160Da (also known as AS160 and TBC1D1) is the Rab GTPase-activating protein and a key regulator of insulin-stimulated glucose uptake, and is expressed in multiple tissues [55]. Site-specific AS160 and TBC1D1 phosphorylation by Akt or other kinases is postulated to change Rab GTPase-activating protein activity and release GLUT4 vesicles. This is an important factor in controlling glucose uptake in the body [56]. Similar to these results, differentiated cells treated with CMRO6 or insulin showed significantly increased phosphorylation of Akt at 473, AS160 at Thr 642, and TBC1D1 at Ser700 compared to control cells. It was confirmed that the CMRO6 stimulates glucose uptake in differentiated cells via phosphorylating insulin-related signaling pathways. There is evidence that MAPKs and ERK1/2 play a role in the regulation of adipocyte differentiation [57]. Recent studies showed that the inhibition of MAPKs and ERK1/2 could result in a significant reduction in differentiation and lipid accumulation in adipocytes [58,59,60]. These findings confirm that both MAPK and ERK1/2 activation are essential for adipogenesis and lipogenesis. We therefore assessed CMRO6 effects on phosphorylation of p38MAPK at Thr180/Tyr182 and Erk44/42 at Thr202/Tyr204 in adipocytes. The outcome of the present study revealed that the cells treated with CMRO6 significantly reduced phosphorylated p38MAPK and ERK1/2 proteins compared to control cells, thus suggesting that CMRO6 treatment inhibits differentiation and lipid accumulation in adipocytes by decreasing the phosphorylation levels of ERK1/2 and p38MAPK, which inhibits transcription factors associated with adipocyte differentiation, such as PPAR-γ, C/EBPα, and C/EBPβ, as well as lipogenic enzymes, such as FAS and ACC. Furthermore, in vivo studies are required to determine the molecular mechanism of CMRO6-regulated fat deposition and glucose uptake through p38MPAK, Erk44/42, and Akt signaling pathways.

## 4. Materials and Methods

### 4.1. Isolation of Bacillus ginsengihumi

*Bacillus ginsengihumi* (CMRO6) was isolated from a whole crop rice sample using HiChrome Bacillus Agar medium (Himedia Laboratories, Maharashtra, India) using a ten-fold serial dilution method. Species identification was performed via biochemical and 16S rRNA sequencing methods (Solgent Co, Seoul, Korea). *Bacillus ginsengihumi* was subcultured in nutrient broth at 37 °C for 24 with mild shaking in an orbital shaker (150 rpm). Fresh culture was preserved in 40% glycerol and stored at −20 °C for short time periods and at −80 ° C for long time periods, for further use.

### 4.2. Microbial Metabolite Production and Lyophilization

Fresh CMRO6 cultures were inoculated into the nutrient broth (BD Difco, New jersey, Franklin Lakes, United States) and incubated for 48 h at 37 °C with mild shaking in an orbital shaker (150 rpm). Cell-free metabolites were obtained by centrifugation at 4000× *g* for 60 min, and the supernatant was then filtered through different filters (Whatman No.1 filter; 1.75 μm and 0.2 μm membranes). The filtered supernatant was lyophilized at −40 °C under less than 50 m Torr pressure (Ilshin Lab. Co., Ltd. Seoul, Korea). A lyophilized powder was sterilized with sun clean bactericide (MY BreeZe mini with sun clean bactericide-#1731, 30000PPM, Mirai Co. Ltd., Chiba, Kashiwa-shi, Japan). The nutrient broth for the control treatment was prepared the same way [15].

### 4.3. Cytotoxic Effects of Cell-Free Metabolites (CMRO6)

Pre-adipocytes (3T3-L1, ATCC-173, Manassas, VA, USA) at a density of 1 × 10^4^/well were seeded in 96-well cell culture plates containing 10% FBS-DMEM 30-2002 medium (FBS-ATCC 30-2020 and DMEM ATCC 30-2002, Manassas, VA, USA) and incubated at 37 °C, 5% CO_2_ for 24 h. After 24 h, the cells were treated with different concentrations of CMRO6 (0.98 µg/mL–500 µg/mL) for another 24 and 48 h under normal cell culture conditions. After that, 10µL of EZ-cytox reagent (DoGenBio, Seoul, South Korea # EZ-1000) was added to each well and incubated for 30 min to 60 min, and optical intensity was measured at a 450 nm (i3 Spectramax (Molecular Device, San Jose, CA, USA [15].

### 4.4. Induction of Lipid Accumulation in 3T3-L1 Adipocytes

The 3T3-L1 pre-adipocytes (ATCC CL-173, ATCC, Manassas, VA, USA) were seeded with a density of 1.5 × 10^4^ and 3.0 × 10^4^/well into 12- and 6-well culture plates, respectively, and incubated at 37 °C, 5% CO_2_. After 48 h of confluence, differentiation cocktails (1 µg/mL insulin, 0.5 mM IBMX in 0.5 M KOH, and 1 μM dexamethasone in ethanol, Sigma-Aldrich, Saint Louis, MI, USA) in 10% FBS-DMEM were used to induce lipid accumulation and differentiation in 3T3-L1 adipocytes. Cells were then switched to insulin medium for another 48 h. In order to determine how CMRO6 affects differentiation and lipid accumulation, the cells were treated every two days with CMRO6 in 10% FBS-DMEM from the day differentiation began to the end of the experiment [61].

### 4.5. Detection and Determination of Lipid Accumulation by Oil Red O Stain

The experimental cells were fixed in 10% formalin for 1 h and then washed with 40% isopropyl alcohol thrice. Then, each well was stained with 3 mL Oil Red O stain (0.35% in isopropyl alcohol, Sigma-Aldrich, Saint Louis, MI, USA) and incubated for 15 min at room temperature, followed by three washes with PBS or water. The stained lipid cells were observed under a microscope and photographed (Evos cell image system, Fisher Scientific, Waltham, MI, USA). The stained lipids were then extracted from the experimental cells using 100% isopropyl alcohol and measured at a wavelength of 490 nm. Finally, by comparing the treatment to the control sample, the percentage of lipid content was calculated [15].

### 4.6. Proteins Extraction and Immunoblot Analysis

Experimental proteins were extracted with cell lysis buffer (RIPA, Rockland, Waltham, MI, USA) in the presence of phosphatase and protease inhibitor cocktails (Roche, Basel, Switzerland and Sigma-Aldrich, Saint Louis, MI, USA, respectively). The total protein content was determined using the BCA method (ThermoFisher Scientific, Waltham, MI, USA). Equal amounts (15 μg/well) of experimental proteins were separated by SDS-PAGE (Mini Protean Pre-casting gels, 12% BioRad, Hercules, CA, USA) and the separated proteins were then transferred onto PVDF (polyvinylidene difluoride (PVDF)) membranes using the Turbo Transfer system (BioRad, Hercules, CA, USA). The membranes were immunoblotted with respective primary antibodies at 1:1000 dilutions (Cell Signaling Technology, Danvers, MA, USA). A Western Breeze chemiluminescence kit (WB7106, Invitrogen, Waltham, MI, USA) was used to detect the expression of the protein in experimental samples. ImageJ software version 1.49 (32 bit, Wayne Rasband, National Institute of Health, Bethesda, MD, USA) was used to determine the band intensity of proteins [15].

### 4.7. 2-Deoxy-D-Glucose (2DG) Uptake in Differentiated 3T3-L1

Glucose uptake was measured using a Promega glucose uptake-Glo assay kit based on the protocol provided by the manufacturer (Promega Co. Madison, WI, USA # J1342). The 3T3-L1 cells were grown in the 96-well cell culture plate at a density of 20,000/well. The differentiation was initiated by a differentiation induction medium (insulin, dexamethasone, and IBMX) similar to the above-said protocol. Eight-day differentiated 3T3-L1 cells were treated with CMRO6 or insulin for 2 h under 5% CO_2_ at 37 °C after overnight starvation. Subsequently, the media was replaced with 50 μL of 2DG in PBS and incubated for 10 min at 25 °C. Then, 25 μL of stop buffer was added, mixed well, and neutralized with 25 μL neutralization buffer. Finally, 100 μL of detection reagent was added and incubated at 25 °C for 1 h, and the luminescence was recorded with 0.3–1.0 s integration using a luminometer (SpectraMax i3x Multimode Detector, Molecular Devices, San Jose, CA, USA).

### 4.8. Statistical Analysis

Statistical analysis was performed on experimental data with SPSS16.0 (SPSS version 16.0, SPSS Inc, Chicago, IL, USA). Statistical significance between control and experimental data was determined with a one-way ANOVA and post hoc test, and multivariate comparison with the Duncan test and independent *t*-test, with *p* < 0.05 significance level.

## 5. Conclusions

Cell-free metabolites from *Bacillus ginsengihumi*-RO6 (CMRO6) inhibited lipogenesis and adipogenesis by downregulating the expression of key transcriptional factors, which included PPAR-γ, C/EBPα, C/EBPβ, and SREBP-1, and the key lipogenic enzymes, such as FAS and ACC. CMRO6 inhibits the expression of adipogenic- and lipogenic-related genes in adipocytes by downregulating phosphorylated p38MAPK and Erk44/42 expression. Furthermore, CMRO6 induced significant increases in glucose uptake through increasing phosphorylated Akt, AS160, and TBC1D1 expression, as well as increasing adiponectin levels and decreasing FABP4 levels. In conclusion, the overall results suggest that CMRO6 from *Bacillus ginsengihumi* has the potential to prevent lipid accumulation as well as improve glucose uptake by modulating p38MPAK, Erk44/42, and Akt signaling pathways. Despite this, deeper investigation is still needed to clarify the potential role of *Bacillus ginsengihumi* in the gastrointestinal tract as a probiotic.

## Data Availability

The experimental data are available on request by corresponding author.
